# Research trends and hotspots of COVID-19 impact on sexual function: A bibliometric analysis based on Web of Science

**DOI:** 10.3389/fpubh.2022.976582

**Published:** 2022-08-19

**Authors:** Xiaodu Xie, Pan Lei, Lumiao Liu, Jian Hu, Peihe Liang

**Affiliations:** ^1^Department of Urology, The Second Affiliated Hospital of Chongqing Medical University, Chongqing, China; ^2^Department of Anesthesiology, The Second Affiliated Hospital of Chongqing Medical University, Chongqing, China

**Keywords:** COVID-19, bibliometrics, Web of Science, sexual function, hotspots

## Abstract

**Background:**

The outbreak of coronavirus disease 2019 (COVID-19) has brought indelible harms to the world and aroused great concern worldwide. This paper aims to analyze the impact of COVID-19 on sexual function using bibliometrics, and summarize research hotspots in this field.

**Methods:**

Relevant publications concerning the impact of COVID-19 on sexual function in the Web of Science collection database (WoSCC) between January 1, 2020 and March 12, 2022 were screened and analyzed by bibliometric analysis using the visualization software CiteSpace and VOSviewer.

**Results:**

Of the 1,054 publications screened, the United States (US) contributed the most (398/37.8%), followed by the United Kingdom (UK) (119/11.3%). Among all institutions, the University of Toronto in Canada enjoyed the largest number of publications (30), and Johns Hopkins University in the US enjoyed the highest frequency of citation (235). The journal *INTERNATIONAL JOURNAL OF ENVIRONMENTAL RESEARCH AND PUBLIC HEALTH* published the largest number of studies in this field (31), and the most-cited journal was *LANCET*. “Chow, Eric,” “Ong, Jason J,” and “Stephenson, Rob” tied for first place in publications (8), and “Fish, Jessica N.” enjoyed the highest number of citations (99). Burstness analysis of references and keywords showed that the developing research trends in this field mainly focused on “sexual transmission” and “angiotensin converting-enzyme 2 (ACE2)” during the COVID-19 pandemic.

**Conclusion:**

The impact of COVID-19 on sexual function remains an urgent concern at present, and the management of sexual health during the pandemic needs to be further improved. More frequent and deeper cooperation between countries and institutions is required in future. Meanwhile, searching for more evidence on whether COVID-19 can achieve sexual transmission and the pathophysiological mechanisms underlying the impact of COVID-19 on sexual function remains a focus of research in the coming years.

## Introduction

Novel coronavirus pneumonia (NCP) is an acute infectious pneumonia with respiratory symptoms, which was declared an international public health emergency by the World Health Organization (WHO) on January 30, 2020 (1). The WHO named this virus “2019 novel coronavirus” (2019-nCoV), and on February 11, 2020, the NCP infected by 2019-nCoV was officially named “coronavirus disease 2019” (COVID-19). The clinical symptoms associated with COVID-19 range from fever and dry cough to acute respiratory distress syndrome (ARDS) and septic shock. In addition to lung lesions, COVID19 can cause damage to the kidney, heart, brain and other organs, and induce multiple organ dysfunction syndrome (MODS) (2).

However, outbreaks like COVID-19 can also affect sexual and reproductive health and rights in many ways at the individual, systemic and societal levels (3). Sexual function, as an important guarantee for the quality of life, is deeply affected by a variety of factors, including overall mental health. Despite the rigorous precautions, COVID-19 has escalated to pandemic levels, leading to increasing global depression, exhaustion, and potentially long-term negative effects on mental and sexual health. Additionally, existing studies have disclosed that patients with COVID-19 can experience male sexual abnormalities such as testicular and penis shrinkage, decreased libido, erectile dysfunction, and hypogonadism (4, 5). Clinical experience from previous pandemics has shown few clinical manifestations of sexual dysfunction. With a deeper understanding about the COVID-19 pandemic, human reproductive health associated with COVID-9 infection has gradually attracted increasing attention. Given the significance of sexual health in promoting community fertility and health, it seems extremely necessary to study the field of sexuality during the pandemic.

In recent years, bibliometrics has gradually become prevalent, which takes the literature system and bibliometric features as the research object, and adopts mathematics and statistics to quantitatively analyze publications related to the research content and reveal the trends and hot spots in this field. The number of publications on COVID-19 is increasing, but few bibliometric studies are concerned with COVID-19. To the best of our knowledge, no bibliometric analysis has specifically discussed the impact of COVID-19 on sexual function. In this paper, we used VOSviewer and CiteSpace to perform a multidimensional statistical analysis of the literature on the effect of COVID-19 on sexual function in the WOS database between January 1, 2020, and March 12, 2022, hoping that the results obtained could provide valuable references for future related study.

## Materials and methods

### Data acquisition and search strategies

**Figure 1:**
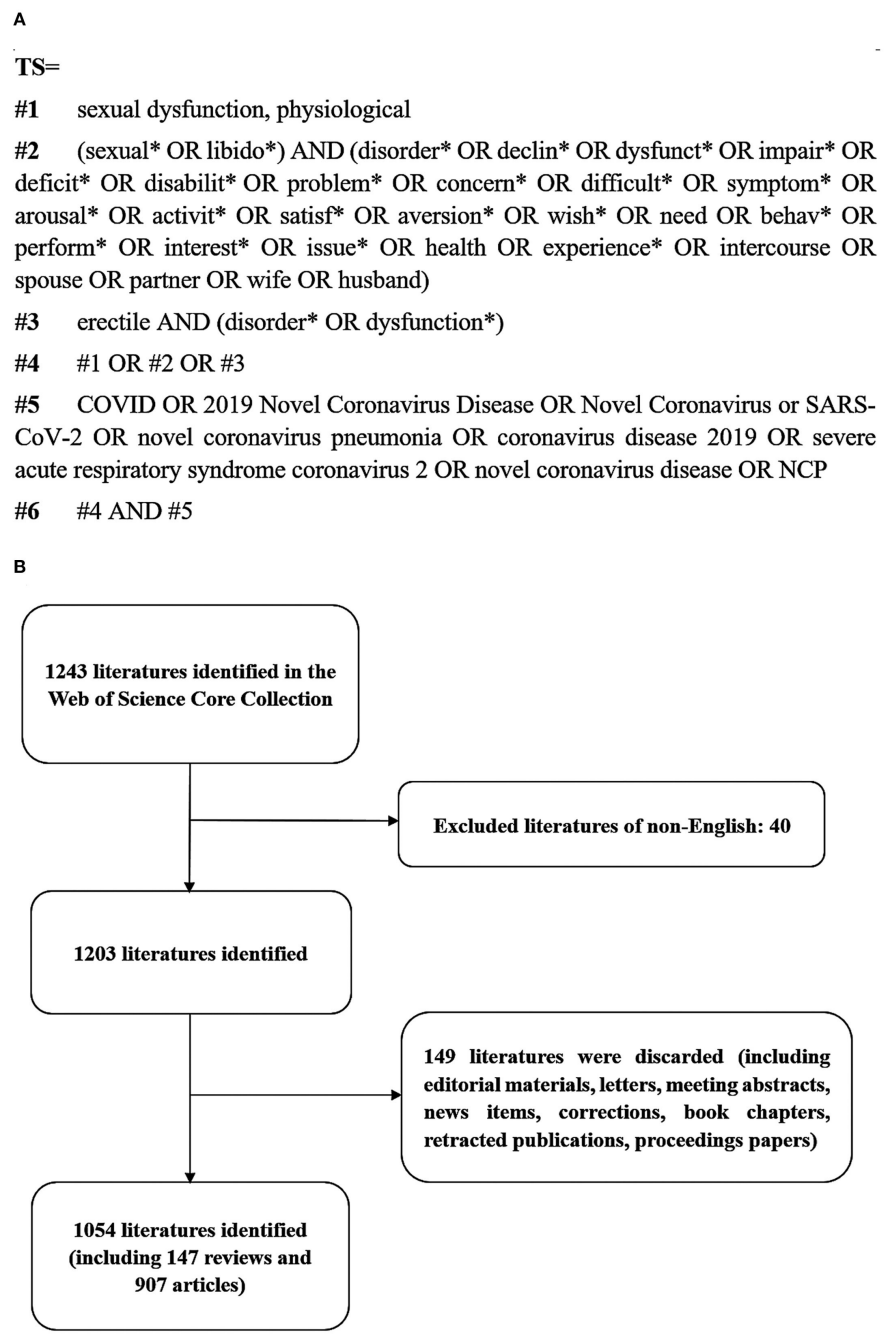
Flowchart of the search strategy and screening process. **(A)** Retrieval strategy. **(B)** Screening process. *, Truncation operator; #, connection character.

Web of Science (WoS) is the world's largest comprehensive academic information resource covering the largest number of disciplines, including more than 8,700 core academic journals. In this study, we collected and analyzed the data by searching the Science Citation Index Expanded Web of Science Core Collection (WoSCC). To avoid omissions due to frequent updates of the database, the literature retrieval and data download were completed within 1 day, ranging in time from January 1, 2020 to March 12, 2022. The search strategy and screening process are shown in Figure 1. Only original articles and reviews published in English were included in this paper. The search process was conducted independently by two individuals, and any disagreement would be consulted with a third experienced corresponding author before making the final decision.

### Data collection

Two authors (Xiao-du Xie and Pan Lei) independently screened and extracted all required data from the included articles, including the titles, authors and their H-Index, keywords, journals, countries, institutions, citations and references. The obtained data were analyzed by CiteSpace version 6.1.R2, Microsoft Excel 2019, and VOSviewer 1.6.18.0.

### Bibliometric analysis

We intended to conduct the analysis by sorting out the data characteristics of all the relevant literature. Knowing that Journal Citation Reports (JCR) is one of the important indicators to measure the value of scientific research, we used the Impact Factor (IF) from JCR published in 2020 in this study. The H-index is also one of the most widely used academic indicators to measure the output and influence of researchers. The higher a person's H-Index means the greater impact of his/her papers. Therefore, the H-Index can accurately reflect a person's academic achievements (6).

All analysis in this study were mainly performed by Microsoft Excel 2019, CiteSpace version 6.1.R2 and VOSviewer 1.6.18.0. Excel was used to make histograms and pie charts for data visualization. Both VOSviewer and CiteSpace are developed based on bibliometric methods to map knowledge graphs in various fields. VOSviewer is a statistical software that can utilize the links between nodes to identify bibliometric characteristics to further provide references in the field, such as countries, organizations, journals, researchers, or keywords (7). We used VOSviewer software to map collaborative networks (including countries, institutions, and authors), co-citation networks (including journals and references), and keyword co-occurrence networks. For all the contents analyzed by VOSviewer, we chose the fractional counting method, which means the weight of a link is fractionalized. In co-authorship analysis of countries/institutions/authors, the minimum number of documents of a country was set to 10/5/3, with the minimum number of citations set to 0. In co-citation analysis of journals and references, we included documents occurring over 20 times into analysis. In co-occurrence analysis of keywords, we selected author keywords for analysis, with the minimum number of occurrences of a keyword set to 5.

CiteSpace is another visualization analysis software developed by Professor Chen Chaomei, which focuses on analyzing the potential knowledge contained in the scientific literature (8). Compared with VOSviewer, CiteSpace allows for better exploration of research advances and current research frontiers in a subject area, and finally discerns the emerging trends of this field (9). In our study, we employed CiteSpace to conduct co-citation analysis (along with cluster analysis) and burstness analysis of references and keywords, which is helpful in complementing and grasping the research direction (10). The minimum burst duration was set to 1 year. Time slicing was performed from January 2020 to December 2022, with 1 year per slice. The selection criteria were that top 50 levels of most cited or occurring items from each slice were selected for analysis, and other parameters were set to default values. To identify important pivot points within a field, we further calculated nodes with high centrality (>0.1) in references and keywords. The “compute node centrality” function in the menu was manually clicked if the number of network nodes was >350.

## Results

### Contributions of countries and regions to global publications

**Figure 2:**
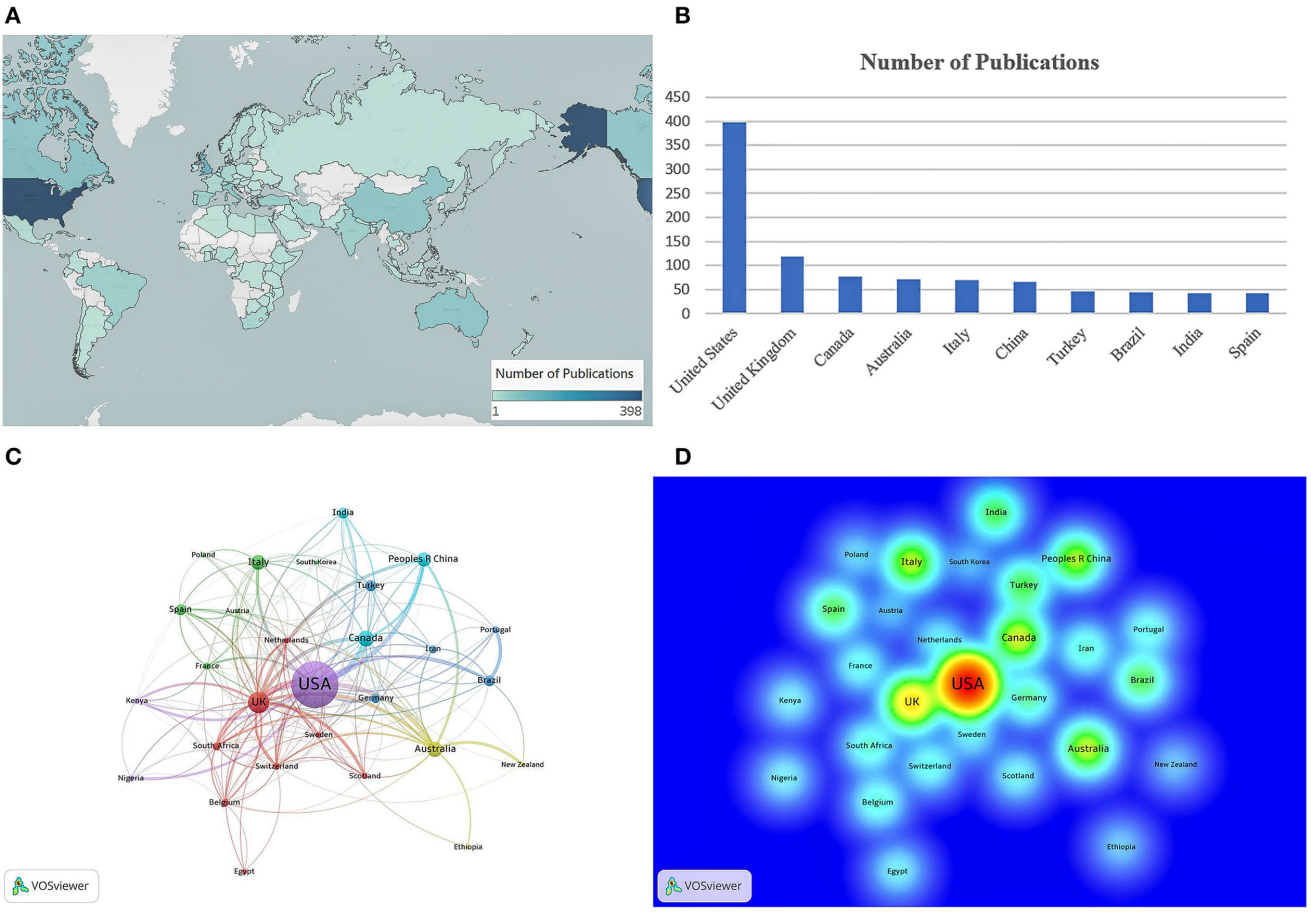
Contributions of different countries and regions to publications. **(A)** Geographic map of research countries and regions. **(B)** Histogram of top 10 countries in terms of the number of documents. **(C)** Cooperation network visualization between countries and regions. **(D)** Density map of countries and regions.

A total of 1,054 publications (907 articles and 147 reviews) from January 1, 2020 to March 12, 2022 met the inclusion criteria. The geographic map of research countries and regions was mapped using the Tableau Public software (https://public.tableau.com/s/) (Figure 2A). The US ranked first in terms of the number of publications with 398 articles (37.8%), followed by the UK with 119 (11.3%), Canada with 77 (7.3%), Australia with 71 (6.7%), and Italy with 69 (6.5%) (Figure 2B). The cooperation network between countries and regions is shown in Figure 2C. The size of the circles indicates the number of publications in each country or region, and the number and width of the lines between the circles represent the degree of cooperation. The US enjoyed the highest degree of aggregation and the strongest total link strength in the network, indicating the dominance of its influence in this field, followed by the UK and Canada. The density map in Figure 2D can reflect more intuitively that publishing centers are mainly distributed in the US, Europe, and other countries and regions.

### Analysis of institutional distribution

**Figure 3:**
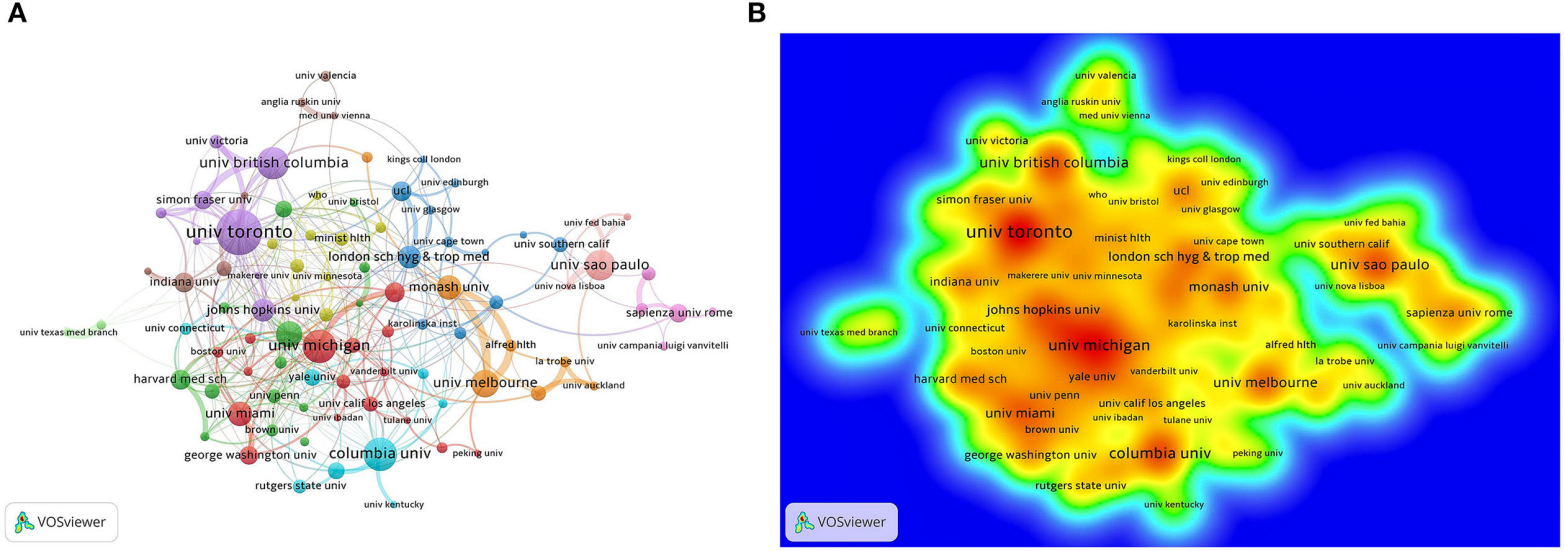
Contributions of different institutions to publications. **(A)** Cooperation network visualization between institutions. The size of the node indicates the number of publications and different colors represent different clusters. **(B)** Density map of institutions.

The Canadian University of Toronto was the institution with the most publications (*n* = 30) with the strongest total line strength in the institutional collaboration network (Figure 3A). The distribution of institutional contributions is presented in Figure 3B, where red represents more contribution and green represents less contribution. The top three institutions were the University of Toronto, Columbia University, and the University of Michigan. The top 15 institutions with the most publications are shown in Table 1. Of the top 15 institutions, eight were located in the US, with the most cited institution being Johns Hopkins University (235 citations); two in Canada; two in Australia; two in the UK and one in Brazil, which further demonstrates the dominance of the US in this aspect.

**Table 1:** Top 15 institutions in terms of the number of publications.

**Rank**	**Institution**	**Number of publications**	**Citations**
1	University of Toronto, Canada	30	61
2	Columbia University, USA	22	186
3	University of Michigan, USA	22	99
4	University of British Columbia, Canada	21	56
5	University of São Paulo, Brazil	20	112
6	University of Melbourne, Australia	18	182
7	University of North Carolina, USA	17	70
8	Monash University, Australia	16	188
9	University of Miami, USA	16	91
10	Johns Hopkins University, USA	15	235
11	London Sch Hyg & Trop Med, UK	15	40
12	Harvard Medical School, USA	13	169
13	Indiana University, USA	13	143
14	University College London, UK	13	126
15	University of California, San Francisco, USA	13	61

### Author collaboration network

**Figure 4:**
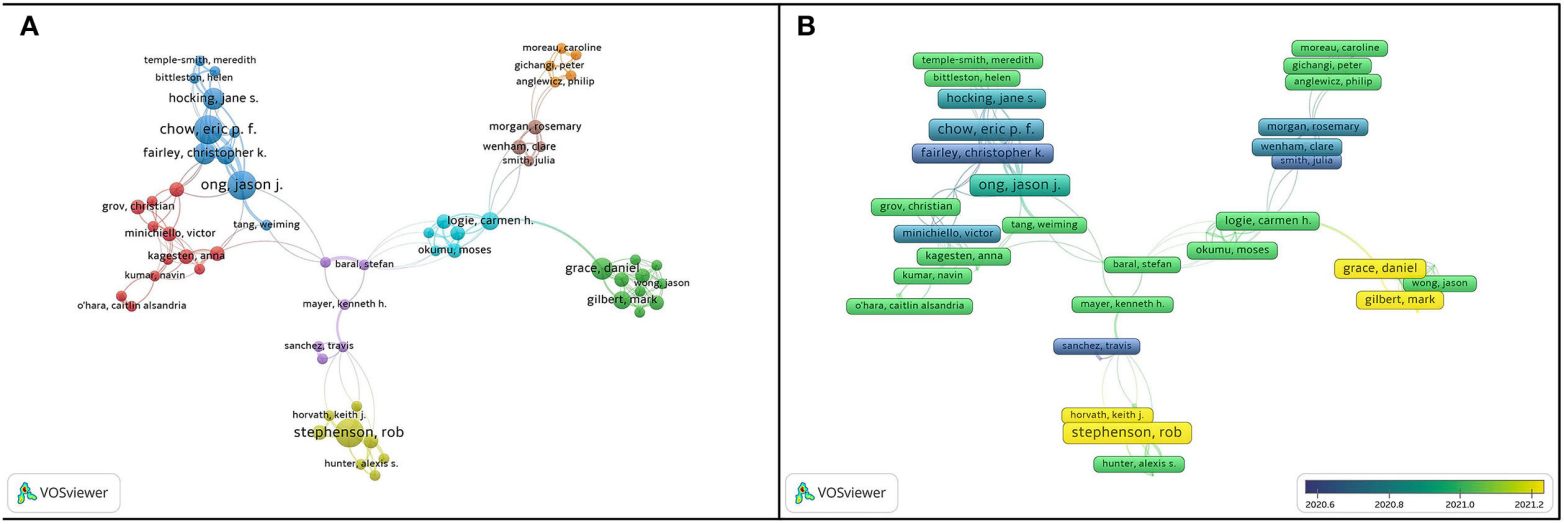
Visualization map of research authors. **(A)** Cooperation network visualization of authors. **(B)** Overlay visualization map based on the weights of documents.

Among the 5,194 authors involved in the publications, 171 authors with three or more publications were selected to produce a collaborative network, and the largest set of connected items, containing 61 researchers, was selected as shown in Figure 4A. The size of the nodes represents the number of publications, and the links between the nodes represent the collaboration between researchers. The collaboration network is relatively scattered, forming several independent clusters, represented by “Chow, Eric,” and “Stephenson, Rob”. Figure 4B discloses a trend of publications by gradient colors (the closer to yellow means the nearer time of publication). Most authors had fewer relevant publications recently. Some authors like “Stephenson, Rob,” “Grace, Daniel,” and “Gilbert, Mark,” had some new papers in the past 6 months. For example, on January 19, 2022, Stephenson pointed out that with the progress of the COVID-19 pandemic, the sexual behavior among homosexuals, bisexuals, and other men who had sex with men had declined significantly (11).

To make the data more intuitive and concrete, we list the information about the top 10 authors in terms of publications in Table 2. “Chow, Eric,” “Ong, Jason J,” and “Stephenson, Rob” tied for first place, with eight articles each. Six of the 10 authors are from Australia, three from the US, and one from Canada, proving that the achievements of Australia deserve our close attention in this field. It is worth noting that “Fish, Jessica N.” of the US had the highest number of citations (*n* = 99). Although he only has published six articles, his views in the field have attracted widespread attention. For instance, on September 14, 2020, Fish, Jessica N. highlighted that lesbian, gay, bisexual, transgender, and queer/questioning (LGBTQ) adolescents were more likely to have poorer mental health and wellbeing compared with their non-LGBTQ peers. Concerted efforts are required to provide LGBTQ youth with the necessary resources and support during the COVID-19 pandemic.

**Table 2:** Top 10 authors ranked by article counts.

**Rank**	**Author**	**Country**	**Institution**	**Article counts**	**Citations**	**H-Index**
1	Chow, Eric	Australia	Monash University	8	93	37
2	Ong, Jason J.	Australia	University of Melbourne	8	62	10
3	Stephenson, Rob	USA	University of Michigan	8	62	31
4	Maher, Lisa	Australia	University of New South Wales Sydney	6	72	42
5	Grace, Daniel	Canada	University of Toronto	6	5	12
6	Fairley, Christopher K.	Australia	University of Melbourne	6	40	66
7	Hocking, Jane S.	Australia	University of Melbourne	6	91	46
8	Fish, Jessica N.	USA	University of Maryland	6	99	18
9	Ramasamy, Ranjith	USA	University of Miami	6	40	29
10	Bourne, Adam	Australia	La Trobe University	5	55	20

### Analysis of journal distribution

**Figure 5:**
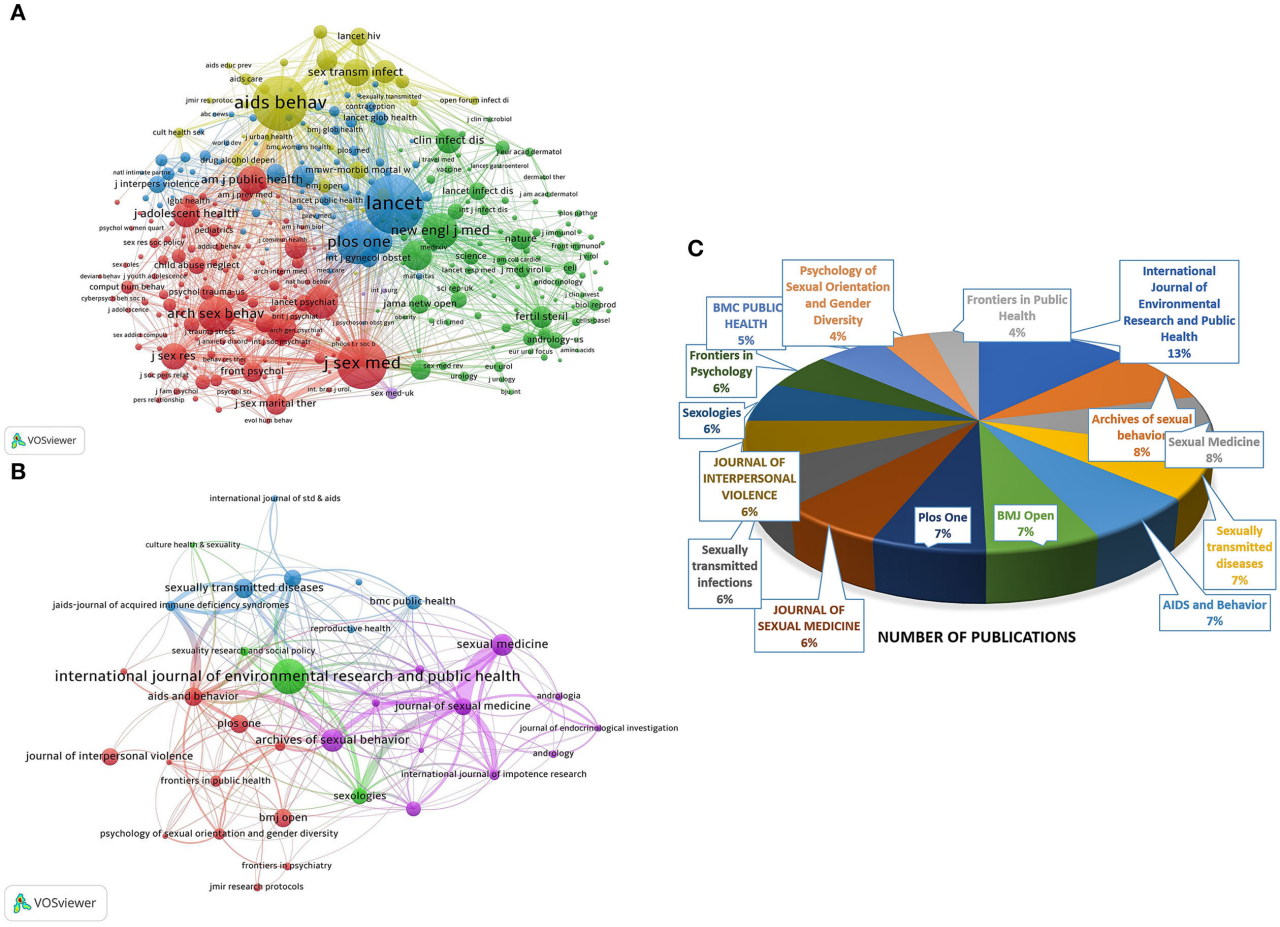
Visualization map of journals. **(A)** Co-citation network of journals based on reference sources. **(B)** Cross-citation analysis of journals. **(C)** Pie chart of top 15 journals that published the largest number of documents.

For the co-citation network of journals, we set the minimum number of citations to 20 and visualized 331 journals of the 14,631 sources (Figure 5A). The size of the nodes represents the citations of each journal, and the lines between the nodes represent the link strength. As shown in Figure 5A, of the four clusters included, *LANCET* (IF = 202.731), *AIDS AND BEHAVIOR* (IF = 4.852), *THE JOURNAL OF SEXUAL MEDICINE* (IF = 3.937), *PLOS ONE* (IF = 3.752), and *THE NEW ENGLAND JOURNAL OF MEDICINE* (IF = 176.079) ranked among the top five citations.

Figure 5B shows further exploration of the relationship between journals in the field by making a cross-citation network. The thickness of the lines between nodes means the strength of mutual citations between journals. *THE JOURNAL OF SEXUAL MEDICINE* and *SEXUAL MEDICINE* had the strongest relationship. Additionally, Figure 5C and Table 3 show the top 15 journals in this field by publications to concretize the data of the cross-citation network. The journal with the most publications is *INTERNATIONAL JOURNAL OF ENVIRONMENTAL RESEARCH AND PUBLIC HEALTH* (IF = 4.614), with 31 publications (13%), which were cited 112 times. *ARCHIVES OF SEXUAL BEHAVIOR* (IF = 4.891) ranked second, with 20 records (8%) that were cited 29 times. It is noteworthy that *AIDS AND BEHAVIOR* (IF = 4.852) and *THE JOURNAL OF SEXUAL MEDICINE* (IF = 3.937) had the highest number of citations, accounting for 333 and 289, respectively, although they ranked 5th and 8th in publications respectively.

**Table 3:** Top 15 journals ranked by the number of publications.

**Rank**	**Journal**	**Number of publications**	**Impact factor (2021)**	**Quartile in category (2021)**	**Citations**
1	International Journal of Environmental Research and Public Health	31	4.614	Q2	112
2	Archives of Sexual Behavior	20	4.891	Q1	29
3	Sexual Medicine	19	2.523	Q3	62
4	Sexually Transmitted Diseases	17	3.868	Q3	93
5	Aids Behavior	16	4.852	Q1	333
6	BMJ Open	16	3.006	Q2	42
7	PLoS ONE	16	3.752	Q2	108
8	Journal of Sexual Medicine	15	3.937	Q2	289
9	Sexually Transmitted Infections	15	4.199	Q3	94
10	Journal Interpersonal Violence	15	2.621	Q2	41
11	Sexologies	14	NA	NA	40
12	Frontiers in Psychology	13	4.232	Q1	106
13	BMC Public Health	13	4.135	Q2	15
14	Psychology of Sexual Orientation and Gender Diversity	10	4.617	Q1	40
15	Frontiers in Public Health	10	6.461	Q1	8

NA, not applicable.

### Co-citation network of references

**Figure 6:**
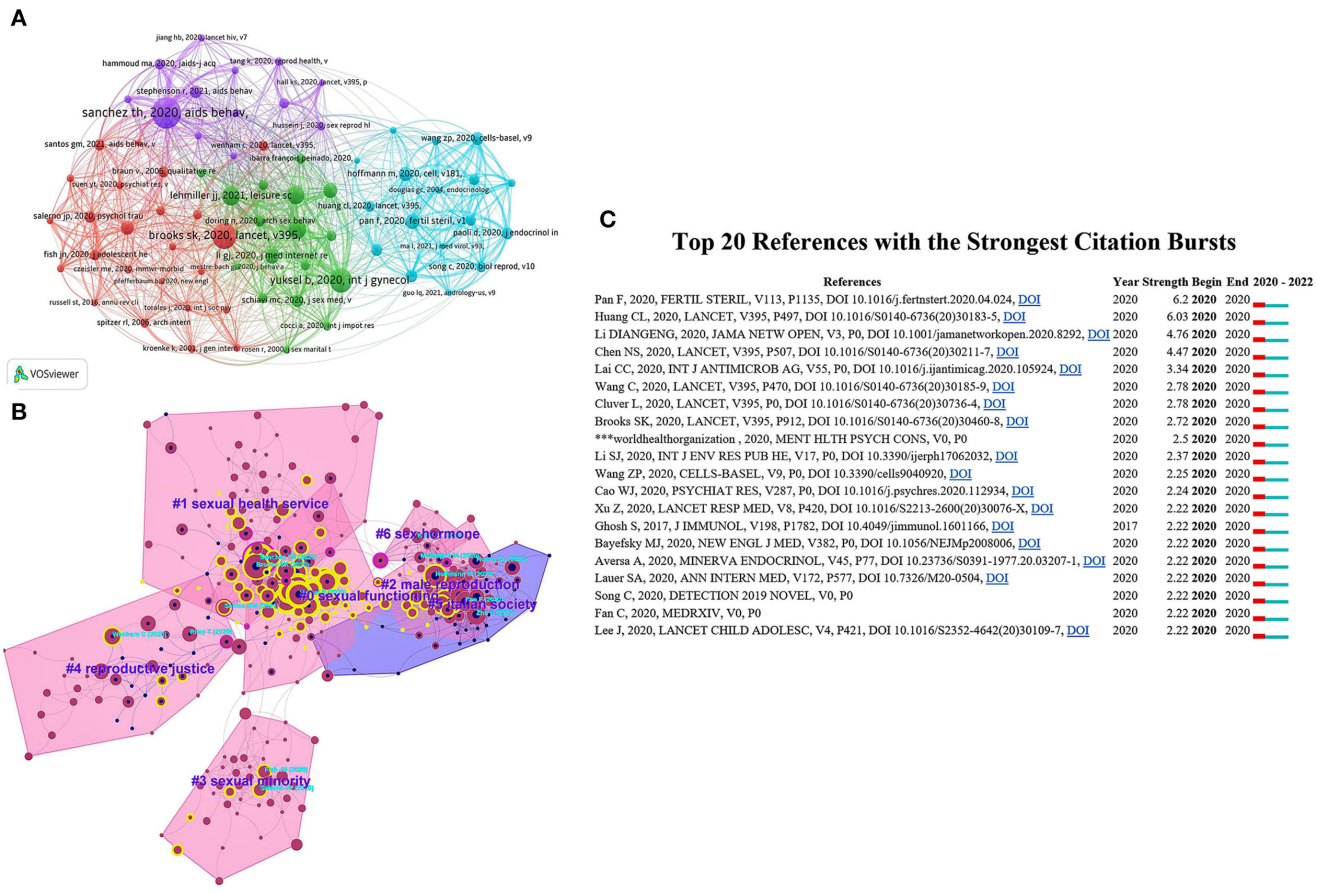
Network visualization map of cited references. **(A)** Co-citation network of references. **(B)** Cluster analysis of references. **(C)** Top 20 references with the strongest citation bursts. ***, Ellipsis.

We analyzed the references of the included papers and constructed a co-citation network (Figure 6A), which was divided into seven co-citation clusters (Figure 6B). Of the 37,402 cited references, the authors set the minimum number of citations of a cited reference at 20, and 71 articles met the threshold. Tables 4, 5 show the top 10 most cited references about frequency and centrality, which mainly involves the significant changes in mental health and sexual behavior of heterosexuals or homosexuals during the COVID-19 pandemic, along with corresponding potential challenges (such as HIV prevention). In terms of frequency, the most cited paper was *Characterizing the Impact of COVID-19 on Men Who Have Sex with Men Across the United States in April, 2020*, which was published by “Travis H. Sanchez”, and this article was cited 98 times. In this article, Travis H. Sanchez elucidated the health inequities and challenges among vulnerable minorities during the COVID-19 pandemic, including men who had sex with men (MSM) (12). In terms of centrality, the article ranking first (centrality = 0.25) was *Impact of sex and gender on COVID-19 outcomes in Europe*, which was performed by Catherine Gebhard. In this review, the author emphasized the need to understand the impact of the sex and gender disparities on the incidence and case-fatality of the corresponding disease in the COVID-19 pandemic, so that appropriate measures could be taken for prevention and treatment (13). The clusters were listed from 2020 to 2022: “sexual functioning” (Cluster 0, *n* = 75), “sexual health service” (Cluster 1, *n* = 62), “male reproduction” (Cluster 2, *n* = 53), “sexual minority” (Cluster 3, *n* = 42), “reproductive justice” (Cluster 4, *n* = 40), “italian society” (Cluster 5, *n* = 34), and “sex hormone” (Cluster 6, *n* = 15). The most relevant citer to the largest cluster 0 was *Sexual health (excluding reproductive health, intimate partner violence and gender-based violence) and covid-19: a scoping review*, which called on researchers to focus efforts on low and middle-income countries (LMICs) and under-researched topics within sexual health (14).

**Table 4:** Top 10 studies ranked by the total citations.

**Rank**	**Title**	**Corresponding authors**	**Journal**	**Publication year**	**Impact factor (2021)**	**Total citations**
1	Characterizing the Impact of COVID-19 on Men Who Have Sex with Men Across the United States in April, 2020	Travis H. Sanchez	Aids and Behavior	2020	4.852	98
2	The psychological impact of quarantine and how to reduce it: rapid review of the evidence	Samantha K Brooks	Lancet	2020	202.731	81
3	Effect of the COVID-19 pandemic on female sexual behavior	Bahar Yuksel	International Journal of Gynecology & Obstetrics	2020	4.447	75
4	Less Sex, but More Sexual Diversity: Changes in Sexual Behavior during the COVID-19 Coronavirus Pandemic	Justin J. Lehmiller	Leisure Sciences	2020	5.008	61
5	Challenges in the Practice of Sexual Medicine in the Time of COVID-19 in the United Kingdom	Lee Smith	Journal of Sexual Medicine	2020	3.937	60
6	Challenges in the Practice of Sexual Medicine in the Time of COVID-19 in China	Sen Yang	Journal of Sexual Medicine	2020	3.937	58
7	No evidence of severe acute respiratory syndrome-coronavirus 2 in semen of males recovering from coronavirus disease 2019	James M. Hotaling	Fertility and Sterility	2020	7.49	48
8	Impact of the COVID-19 Pandemic on Partner Relationships and Sexual and Reproductive Health: Cross-Sectional, Online Survey Study	Yunxia Cao	Journal of Medical Internet Research	2020	7.076	48
9	SARS-CoV-2 Cell Entry Depends on ACE2 and TMPRSS2 and Is Blocked by a Clinically Proven Protease Inhibitor	Markus Hoffmann	Cell	2020	66.85	45
10	scRNA-seq Profiling of Human Testes Reveals the Presence of the ACE2 Receptor, A Target for SARS-CoV-2 Infection in Spermatogonia, Leydig and Sertoli Cells	Xiaojiang Xu	Cells	2020	7.666	41

**Table 5:** Top 10 cited references by the highest centrality.

**Rank**	**Title**	**Corresponding authors**	**Journal**	**Publication year**	**Impact factor (2021)**	**Centrality**
1	Impact of sex and gender on COVID-19 outcomes in Europe	Catherine Gebhard	Biology of Sex Differences	2020	8.811	0.25
2	Characterizing the Impact of COVID-19 on Men Who Have Sex with Men Across the United States in April, 2020	Travis H. Sanchez	AIDS and Behavior	2020	4.852	0.21
3	Multidisciplinary research priorities for the COVID-19 pandemic: a call for action for mental health science	Emily A Holmes	Lancet Psychiatry	2020	77.056	0.18
4	Clinical course and risk factors for mortality of adult inpatients with COVID-19 in Wuhan, China: a retrospective cohort study	Fei Zhou	Lancet	2020	202.731	0.16
5	Epidemic of COVID-19 in China and associated Psychological Problems	Md Zahir Ahmed	Asian Journal of Psychiatry	2020	13.89	0.15
6	Genomic characterization and epidemiology of 2019 novel coronavirus: implications for virus origins and receptor binding	Roujian Lu	Lancet	2020	202.731	0.14
7	Prevalence and predictors of PTSS during COVID-19 Outbreak in China Hardest-hit Areas: Gender differences matter	Weizhi Liu	Psychiatry Research	2020	11.225	0.13
8	Sex in the Time of COVID-19: Results of an Online Survey of Gay, Bisexual and Other Men Who Have Sex with Men's Experience of Sex and HIV Prevention During the US COVID-19 Epidemic	Rob Stephenson	AIDS and Behavior	2021	4.852	0.12
9	Economic, Mental Health, HIV Prevention and HIV Treatment Impacts of COVID-19 and the COVID-19 Response on a Global Sample of Cisgender Gay Men and Other Men Who Have Sex with Men	Glenn-Milo Santos	AIDS and Behavior	2021	4.852	0.12
10	Contrasting the Perceived Severity of COVID-19 and HIV Infection in an Online Survey of Gay, Bisexual, and Other Men Who Have Sex With Men During the U.S. COVID-19 Epidemic	Rob Stephenson	American Journal of Mens Health	2020	2.403	0.11

In addition, the top 20 references with the strongest citation bursts are listed in Figure 6C. Among them, Pan F, 2020, FERTIL STERIL, V113, P1135 (15) has the highest burst strength (*n* = 6.2), entitled *No evidence of severe acute respiratory syndrome-coronavirus 2 in semen of males recovering from coronavirus disease 2019*. This observational study demonstrated that SARS-CoV-2 was not likely to exist in the semen of males recovering from COVID-19, but the long-term impacts of SARS-CoV-2 on male reproductive function remain to be further investigated.

### Analysis of keywords

#### Co-occurrence network

**Figure 7:**
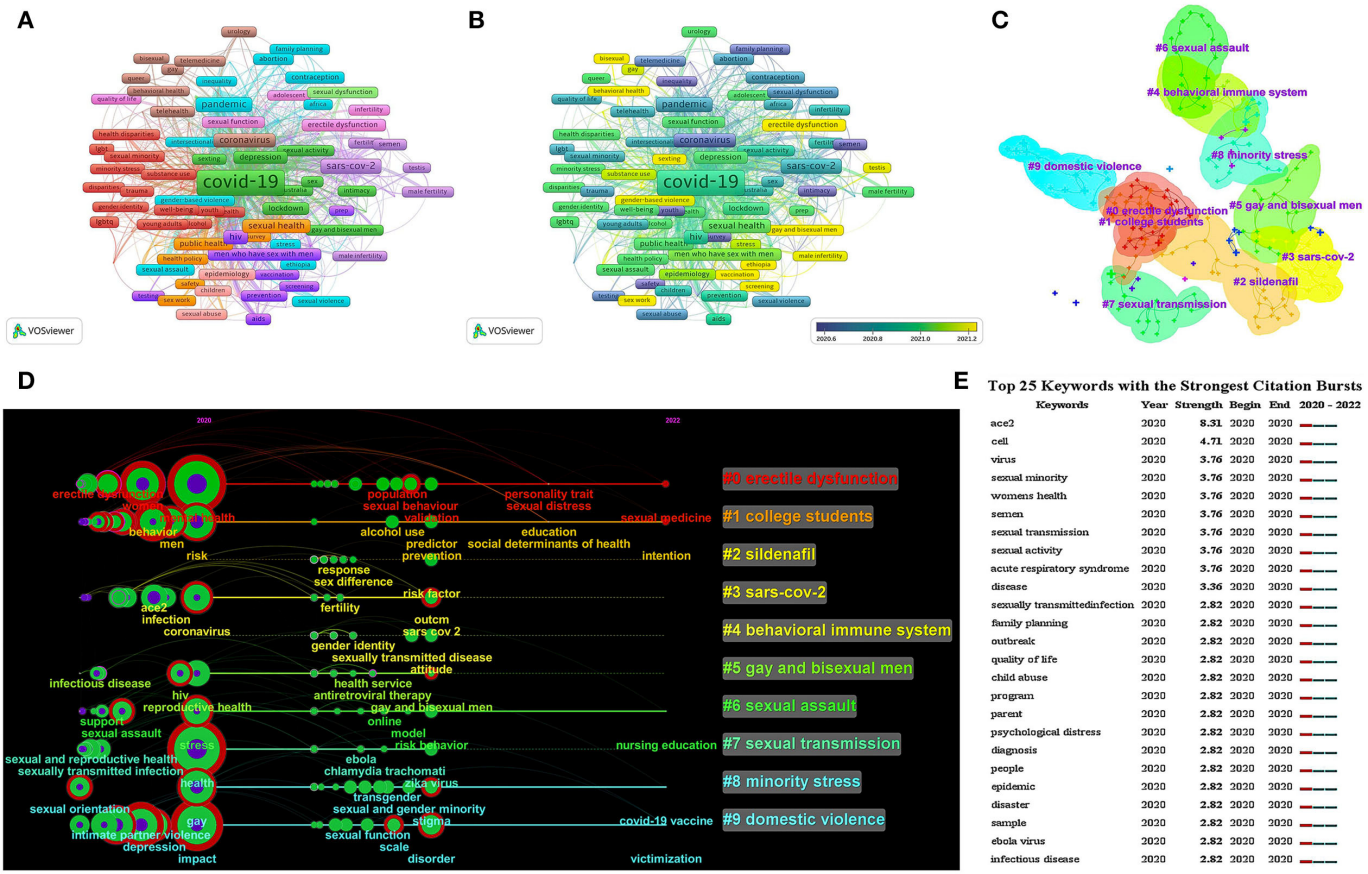
Visualization map of keywords in studies. **(A)** Co-occurrence network of keywords. **(B)** Overlay visualization map based on the occurrences of keywords. **(C)** Cluster analysis of keywords. Different patterns represent a cluster. **(D)** Timeline cluster map of keywords from January 1, 2020, to March 12, 2022. **(E)** Top 25 keywords with the strongest citation bursts.

Keywords represent the core content and main theme of documents. Co-occurrence analysis of keywords can help capture the hot topics in this field. The threshold was that the minimum number of occurrences of a keyword was at 5 times. Of the 2,285 keywords, a total of 138 keywords were identified (Figure 7A). Table 6 shows the top 10 keywords about frequency and centrality. We found that “mental health” was the most popular keyword (72 times) by the number of occurrences, after removing the “COVID-19” (603 times), “SARS-CoV-2” (90 times), and “pandemic” (80 times). Combined with centrality, the other popular keywords were “sexual behavior,” “HIV,” “depression,” “domestic violence,” “erectile dysfunction,” “public health,” “Lesbian” and “sexual dysfunction.” Figure 7B shows the degree to which keywords appear sequentially over time. The color dark green means an earlier occurrence, and yellow indicates a more recent one. The keywords that appeared in the past year mainly include “behavioral health,” “gender-based violence,” “erectile dysfunction,” “sex work,” “male infertility,” “testis,” and “gay and bisexual men.”

**Table 6:** Top 10 keywords by the occurrences and centrality.

**Rank**	**Occurrences**	**Keyword**	**Rank**	**Centrality**	**Keyword**
1	603	COVID-19	1	0.35	Depressive symptom
2	90	SARS-CoV-2	2	0.3	Infectious disease
3	80	Pandemic	3	0.28	Expression
4	72	Mental health	4	0.26	Sample
5	61	Coronavirus	5	0.25	Dysfunction
6	58	Sexual health	6	0.22	Erectile dysfunction
7	57	HIV	7	0.22	Public health
8	34	Depression	8	0.18	Lesbian
9	34	Domestic violence	9	0.16	Sexual dysfunction
10	31	Sexual behavior	10	0.15	HIV risk

Moreover, there were 10 clustering patterns in the field, as shown in Figure 7C. Based on the clustering patterns, the authors deployed the software CiteSpace to create a time-line view of keyword clusters to further grasp the research trends in this field (Figure 7D). The color of the labels of clusters is consistent with the color of the timeline. We found that the research hotspots gradually shifted from mental health, sexual health, ACE2, erectile dysfunction, depression, domestic violence, HIV, and sexually transmitted infection to sexual behavior, health service, sex difference, sexual and gender minority, minority stress, and other aspects.

#### Burst keywords analysis

CiteSpace's bursts analysis is an algorithm based on the word frequency growth to detect professional words with a high word frequency change rate and fast growth rate. The top 25 keywords with the strongest citation bursts are presented in Figure 7E. The term with the highest burst strength was “ACE2” (*n* = 8.31), which provided important insights and references for the trend and focus of later study.

## Discussion

COVID-19, as a global epidemic, has attracted worldwide attention since its outbreak. In addition to causing lung damage, the severe acute respiratory syndrome coronavirus 2(SARS-CoV-2) can also affect multiple organs in the body, causing damage with a variety of sequelae. With the emergence of various symptoms such as loss of libido, erectile dysfunction, and premature ejaculation in patients with COVID-19, the impact of COVID-19 on sexual function has gradually become an urgent clinical concern. The advent of the era of big data requires researchers to fully grasp the development of their research fields. Unlike systematic reviews and meta-analyses, we used VOSviewer and CiteSpace software to synthesize the relevant literature in this field to find out the research hotspots and trends of the impact of COVID-19 on sexual function by using bibliometrics in an attempt to provide important references for future study.

With respect to international cooperation in this field, the US occupied the center of the network, taking the lead in number of publications due to the large number of infected people, sufficient funding, sophisticated equipment, and large numbers of professional researchers. Almost all the countries with the largest number of publications are from Europe and North America, which is attributed to the advanced level of Europe and North America in the medical field. In addition, research institutions in the US (such as Johns Hopkins University, Columbia University, and the University of Michigan) also maintain the leading number of publications and citations. These characteristics all indicate the absolute dominance of the US in this field. However, compared with intra-country cooperation, the cooperation among different countries is slightly insufficient; for instance, the window of external cooperation of each country is limited to the central research institutions within the country. Confronted with a sudden global infectious disease, cooperation is an important way to improve the efficiency of scientific research. To help people understand the biological characteristics of new pathogens as soon as possible and then actively deal with the corresponding harm, it is essential to strengthen the cooperation between countries and institutions to attract enough attention from the world.

The most published journals mainly include sexual medicine related journals (such as *ARCHIVES OF SEXUAL BEHAVIOR, SEXUAL MEDICINE*) and general medical journals (such as *INTERNATIONAL JOURNAL OF ENVIRONMENTAL RESEARCH AND PUBLIC HEALTH, BMJ OPEN, PLOS ONE*). The journal *SEXUAL MEDICINE* and *THE JOURNAL OF SEXUAL MEDICINE* had the most cross-citations with a large overlap in the research field, so relevant researchers could pay close attention to the publication trends of these two journals for more literature support. Meanwhile, *LANCET, AIDS AND BEHAVIOR*, and *THE JOURNAL OF SEXUAL MEDICINE* are the top three co-cited journals, all of which are valued by most researchers in this field. These findings imply that later important achievements in this field may be published in the above-mentioned journals, which may be ideal for future researchers to submit for publication. Furthermore, authors such as “Fish, Jessica N.”, “Chow, Eric”, and “Hocking, Jane S.” are among the highest in the number of publications and citations, and have become an influential core group in the field. It is evident that almost all the highly productive and highly cited authors are from Australia and the US, and Australia in particular has played an important role in this field. However, most researchers have less cooperation, with the clusters scattered. Researchers from Asian countries such as China still need to strengthen their cooperation with researchers from Australia and the US.

Combined with the co-citation analysis of references and occurrence analysis of keywords, epidemiological research of sexual activity has received extensive attention. During the COVID-19, the frequency of sexual intercourse has decreased among heterosexuals or homosexuals (16, 17). Especially for fertile couples, the economic and social pressures brought about by the COVID-19 pandemic have seriously affected their wellbeing and fertility expectations, leading to a decline in fertility rates in most countries and regions (18, 19). There may be a baby boom after regression or eradication of the COVID-19 pandemic. Considering that the COVID-19 pandemic may stay with human beings for a long period, relevant governments should take corresponding regulatory measures to address people's sexual health and control the declining fertility rate. In addition, gendered effects caused by sex differences have begun obtaining attention. Existing evidence has pointed out that the COVID-19 pandemic has effects related to sex and gender, including differences between females and males in the incidence and case-fatality of the disease, and differences in social and economic impacts (such as domestic violence, economic and job insecurity, and increased workload) (13). Especially for domestic violence, the increase of domestic violence is a noticeable problem during the COVID-19 pandemic. Gebrewahd et al. (20) reported that the incidence of intimate partner violence against women was 24.6%, among which psychological violence accounted for the highest percentage (13.3%), followed by physical violence (8.3%) and sexual violence (5.3%). Household stress, disruption of social and protective networks, and reduction of sexual and reproductive health services are unneglectable factors that contribute to intimate partner violence (21). Health officials should intervene to prevent the occurrence of these disorders by refining essential services (such as shelters, rape crisis centers, and counseling) and formulating strategies.

Through analysis of the references and keywords about frequency and centrality, along with cluster patterns, we were surprised to find that much attention was focused on minority groups, such as lesbians, gays, or bisexuals. Affected by the COVID-19, many reasons such as isolation, unemployment, declining incomes, and family pressures have made people's mental health an important issue that cannot be ignored. Especially for minority groups, a considerable number of people experienced varying degrees of psychological distress (depression, depression, and anxiety), which was accompanied by a decrease in the frequency of sexual behavior and a reduction in HIV transmission (12). Nevertheless, given their special social relationships and lifestyle patterns, minorities such as homosexuals face greater challenges than ordinary people, mainly in the difficulty of obtaining HIV prevention and other sexual health services, including the necessary medical guidance and supplies (such as condoms, HIV test strips, and anti-HIV drugs) (22–24). Thus, when a major infectious disease occurs, we need to take necessary measures to give adequate social support and medical assistance to minority groups, improve their physical and mental health, and further reduce the occurrence of related diseases.

Moreover, we further explored the research hotspots through analysis of the burst references and the burst keywords. Sexual transmission and the microbiological research of sexual function (mainly ACE2) are currently important research directions. Existing studies have shown that the semen quality is significantly reduced (such as oligospermia or azoospermia) in men infected with COVID-19, but the semen of most patients will not be infected with SARS-CoV-2 (25–28). SARS-CoV-2 was detected only in the semen of a small number of individuals in the acute stage of infection and the recovery period (29–32). Therefore, there is currently insufficient evidence to support the sexual transmission of SARS-CoV-2. The confirmation of the fact that SARS-CoV-2 can be transmitted sexually might also be a critical part of the prevention of SARS-CoV-2 transmission, which needs to be further verified by larger-scale follow-up studies in future. For ACE2, it has been demonstrated that SARS-CoV-2 can invade human cells through the ACE2 receptor (33). In the male reproductive system, ACE2 is widely expressed in Leydig cells, Sertoli cells, spermatogonial cells, vas deferens cells, and smooth muscle and endothelial cells of the corpus cavernosum (34–37). For sexual dysfunction caused by SARS-CoV-2, there are mainly two reasons: firstly, SARS-CoV-2 can stimulate inflammatory response causing vascular endothelial damage (38), and after SARS-CoV-2 infects normal men, it can cause extensive damage to endothelial cell function in the corpus cavernosum, which induces erectile dysfunction (39, 40); secondly, the combination of SARS-CoV-2 and ACE2 causes damage to Leydig cells, resulting in a decrease in testosterone (T) secretion and a negative-feedback increase in luteinizing hormone (LH), which increases the LH/T ratio, eventually leading to symptoms of hypogonadism (such as loss of libido, erectile dysfunction, and testicular atrophy) (41). It is noteworthy that there are still no sufficient data to indicate the impact of COVID-19 on male fertility, although male sperm contains the ligand processing enzymes (ACE1 and ACE2), which provide COVID-19 with a foothold on the sperm surface and contribute to further integration (42). More research is still required in the future to study the reproductive competence of males with COVID-19 infection.

Compared with males, the impact of COVID-19 on females is currently focused on the psychological aspect and domestic violence, which in turn leads to a decrease in the quality of sexual activity and threats to sexual health. Although it is reported in the literature that the SARS-CoV-2 can also affect the sexual and reproductive function of women, the pathophysiological mechanisms are less clear than those in men, which needs further in-depth exploration (43–45).

## Strengths and limitations

Our paper is one of the first bibliometric analyses to assess the publications about the impact of COVID-19 on sexual function. The research status, hotspots, and future trends in the field were illustrated by a systematic analysis of countries, authors, institutions, journals, citations, and other factors. However, given the limitation of the inclusion of original articles and reviews and the search for English-language studies, as well as the constant updating of the database, the results of the analysis may differ slightly from the actual results. The search database is limited to the Web of Science, and more databases are required to be searched for analysis in the future.

## Conclusion

The impact of COVID-19 on human sexual function has received widespread attention, especially in men. The US holds a dominant position in this field and is likely to continue to maintain its lead for some time in future. The cooperation intensity of various countries and regions throughout the world still needs to be strengthened, especially between developing and developed countries. In addition, the search for more evidence on whether COVID-19 can be transmitted through sexual activities and the pathophysiological mechanisms underlying the impact of COVID-19 on sexual function remains a major focus at present, which requires more studies to raise awareness.

## Data availability statement

The original contributions presented in the study are included in the article/supplementary material, further inquiries can be directed to the corresponding author/s.

## Author contributions

PLi had full access to all the data in the study and takes responsibility for the integrity of the data and the accuracy of the data analysis. PLi and XX study concept and design. XX and PLe acquisition of data and drafting of the manuscript. XX, PLe, LL, and JH analysis and interpretation of data and statistical analysis. XX, PLe, and PLi critical revision of the manuscript for important intellectual content. PLi was supervision. All authors contributed to the article and approved the submitted version.

## Funding

This work was supported by the Innovation Program for Chongqing's Overseas Returnees (CX2019146), High-level Medical Reserved Personnel Training Project of Chongqing (the 4th batch), and Research Program of Basic Science and Frontier Technology in Chongqing (cstc2017jcyjAX0435).

## Conflict of interest

The authors declare that the research was conducted in the absence of any commercial or financial relationships that could be construed as a potential conflict of interest.

## Publisher's note

All claims expressed in this article are solely those of the authors and do not necessarily represent those of their affiliated organizations, or those of the publisher, the editors and the reviewers. Any product that may be evaluated in this article, or claim that may be made by its manufacturer, is not guaranteed or endorsed by the publisher.
